# Auxin and cytokinin control formation of the quiescent centre in the adventitious root apex of arabidopsis

**DOI:** 10.1093/aob/mct215

**Published:** 2013-09-22

**Authors:** F. Della Rovere, L. Fattorini, S. D'Angeli, A. Veloccia, G. Falasca, M. M. Altamura

**Affiliations:** Department of Environmental Biology, Sapienza University of Rome, Italy

**Keywords:** Adventitious root apex, *Arabidopsis thaliana*, auxin biosynthesis, auxin transport, cytokinin localization, quiescent centre, root meristemoids, stem endodermis, thin cell layers, *WOX5*

## Abstract

**Background and Aims:**

Adventitious roots (ARs) are part of the root system in numerous plants, and are required for successful micropropagation. In the *Arabidopsis thaliana* primary root (PR) and lateral roots (LRs), the quiescent centre (QC) in the stem cell niche of the meristem controls apical growth with the involvement of auxin and cytokinin. In arabidopsis, ARs emerge *in planta* from the hypocotyl pericycle, and from different tissues in *in vitro* cultured explants, e.g. from the stem endodermis in thin cell layer (TCL) explants. The aim of this study was to investigate the establishment and maintenance of the QC in arabidopsis ARs, *in planta* and in TCL explants, because information about this process is still lacking, and it has potential use for biotechnological applications.

**Methods:**

Expression of PR/LR QC markers and auxin influx (*LAX3*)/efflux (*PIN1*) genes was investigated in the presence/absence of exogenous auxin and cytokinin. Auxin was monitored by the *DR5::GUS* system and cytokinin by immunolocalization. The expression of the auxin-biosynthetic *YUCCA6* gene was also investigated by *in situ* hybridization *in planta* and in AR-forming TCLs from the indole acetic acid (IAA)-overproducing *superroot2-1* mutant and its wild type.

**Key Results:**

The accumulation of auxin and the expression of the QC marker *WOX5* characterized the early derivatives of the AR founder cells, *in planta* and in *in vitro* cultured TCLs. By determination of PIN1 auxin efflux carrier and LAX3 auxin influx carrier activities, an auxin maximum was determined to occur at the AR tip, to which *WOX5* expression was restricted, establishing the positioning of the QC*.* Cytokinin caused a restriction of *LAX3* and *PIN1* expression domains, and concomitantly the auxin biosynthesis *YUCCA6* gene was expressed in the apex.

**Conclusions:**

In ARs formed *in planta* and TCLs, the QC is established in a similar way, and auxin transport and biosynthesis are involved through cytokinin tuning.

## INTRODUCTION

Adventitious roots (ARs) emerge from organs other than the primary root (PR), such as hypocotyls, stems and leaves. They allow plant adaptation to environmental changes (Li *et al.*, 2009), and are crucial for the survival of important crops, e.g. cereals. However, numerous plants fail to differentiate ARs in response to environmental stress, e.g. soil pollution, and this negatively affects their growth potentials. Also the roots formed by *in vitro* cultured explants are adventitious, and adventitious rooting recalcitrance causes failure of cuttings to grow, with economic losses. The model plant *Arabidopsis thaliana* forms ARs. They originate from the hypocotyl pericycle, at the hypocotyl–PR transition zone (Falasca and Altamura, 2003). Usually they are present in a low number, but mutants overproducing ARs, e.g. *superroot2-1* (*sur2-1*), are known in this species (Delarue *et al.*, 1998).

The indeterminate growth of the PR and lateral roots (LRs) is supported by the quiescent centre (QC), which co-ordinates the activity of the surrounding stem cells, with a central role in establishment, maintenance and elaboration of patterns in the apical meristem (Jiang and Feldman, 2005).

In arabidopsis PR and LRs, the QC is positioned in the centre of the stem cell niche, and is formed by four usually non-dividing cells surrounded by rapidly dividing initial cells, from which the derivative cells forming the tissues of the primary body originate. The QC maintains the undifferentiated state in the surrounding initials by local signalling (van de Berg *et al.*, 1997; Sabatini *et al.*, 2003). The PR is embryonic in origin and thus its QC is established in the embryo. In the arabidopsis embryo, the QC is produced by divisions in the upper hypophysis derivative cell (Jiang and Feldman, 2005). The LRs are post-embryonic, being formed by the PR pericycle cells. The QC is established in the LR tip at stage VII of primordium development (Malamy and Benfey, 1997). Moreover, in both PR and LRs, the QC identity is evidenced by the same QC promoter traps, e.g. QC25 and pAGAMOUS LIKE 42 (pAGL42) (Sabatini *et al.*, 1999; Nawy *et al.*, 2005; Della Rovere *et al.*, 2010).

The ARs are post-embryonic as are LRs, but the establishment of their QC is still an open question. Clowes hypothesized (1956) that the QC was a ubiquitous feature of all Angiosperm root meristems, for at least part of their ontogeny. In arabidopsis, it is still unknown when/how the ARs specify the QC *in planta*, and how long they maintain it. Moreover, a different origin of the ARs, i.e. different founder cells in *in vitro* cultured explants with respect to *in planta*, might affect the establishment and/or the maintenance of the QC in the AR meristem.

Auxin transport via PIN-FORMED (PIN) auxin efflux carriers, e.g. PIN1, is necessary for QC positioning in the PR (Friml *et al.*, 2003), and auxin biosynthesis is needed in PR and LR tips (Ljung *et al.*, 2005), with *YUCCA6*, a gene important in tryptophan-dependent indole-3-acetic acid (IAA) biosynthesis (Mashiguchi *et al.*, 2011), possibly involved (Kim *et al.*, 2007). The AUXIN1 (AUX1)-LIKE AUX1 (AUX/LAX) proteins have auxin influx activity (Péret *et al.*, 2012). Also these proteins are important for LR development (Marchant *et al.*, 2002). Moreover, in PR and LRs, transport and biosynthesis of auxin contribute to establishing and maintaining auxin maxima centred in the QC and columella cells (Sabatini *et al.*, 1999; Benková *et al.*, 2003). In contrast, information about auxin transport/biosynthesis in the arabidopsis AR tip is lacking. It is however known that the synthetic auxin α-naphthalene acetic acid (NAA) increases ARs *in planta* (Falasca and Altamura, 2003), and high levels of endogenous IAA are present in AR-overproducing mutants, e.g. *sur2-1* (Delarue *et al.*, 1998).

In the PR, AtWUSCHEL RELATED HOMEOBOX5 (WOX5) is expressed in the QC, with the function of inhibiting differentiation in the initial distal cells (Sarkar *et al.*, 2007). *WOX5* is auxin inducible, acts downstream of auxin distribution (Ding and Friml, 2010) and seems to be involved in maintenance of the auxin maximum at the PR tip (Gonzali *et al.*, 2005). Moreover, auxin has been suggested to specify the LR QC through WOX5 (Ditengou *et al.*, 2008).

Cytokinin also seems to be involved in QC formation, even if its role needs further investigation. For example, in the PR apex of corn, QC removal causes its regeneration, with a concomitant reduction in cytokinin levels in the proximal meristem adjacent to the QC (Feldman, 1979).

Natural and synthetic auxins, applied alone or in combination with low levels of cytokinin, induce ARs in *in vitro* cultured explants of numerous plants, including arabidopsis. In arabidopsis TCLs, i.e. stem inflorescence tissues external to the vascular system, ARs are induced by indole-3-butyric acid (IBA) (10 µm) plus kinetin (Kin) (0·1 µm) (Falasca *et al.*, 2004). The explants are cultured horizontally, epidermal side up, and ARs appear all along their surface and originate from a unique tissue, i.e. the stem endodermis (Falasca *et al.*, 2004). Whether/how/when auxin polar transport is generated in the AR-forming IBA + Kin-cultured TCLs is unknown.

The aim of this study was to determine whether the QC is established in arabidopsis ARs, whether its establishment and maintenance are under auxin and cytokinin control, and whether the QC specification programme is shared by ARs of different origin, i.e. formed either by the hypocotyl pericycle cells *in planta* or by the stem endodermis cells in the TCLs.

To this aim, the activity of PR/LR QC markers, the expression patterns of the IAA-sensitive *DR5::GUS* reporter and of *PIN1* and *LAX3* auxin carriers, and the AR response of the AR-overproducing *sur2-1* mutant were investigated under various auxin/cytokinin treatments, and *YUCCA6* transcription and cytokinin presence were monitored.

The results show that the QC is established in the ARs. Independently of the founder cells, auxin accumulation and *WOX5* expression characterize the early derivative cells involved in AR formation. By the activity of PIN1 and LAX3, an auxin maximum is determined to occur at the primordium tip, to which *WOX5* expression is restricted, positioning the QC. Tip-localized auxin biosynthesis by YUCCA6 and the activity of the *trans*-zeatin riboside are necessary for QC establishment and maintenance.

## MATERIALS AND METHODS

### Plant material and growth conditions

Seeds of *Arabidopsis thaliana* Columbia (Col) and Wassilewskija (Ws) ecotypes, of *QC25::GUS*, *DR5::GUS*, *PIN1::GUS*, *LAX3::GUS*, *pAGL42::GFP* and *pWOX5::GFP* lines (all in the Col background) and of the *sur2-1* mutant (Ws background) were stratified, sterilized and sown on Petri plates according to Airoldi *et al.* (2010), with minor modifications, i.e. full-strength salts of MS (Murashige and Skoog, 1962) and 1 % sucrose (i.e. hormone-free, HF, growth medium). Alternatively, either 10 µm IBA plus 0·1 µm Kin (Sigma-Aldrich; Falasca *et al.*, 2004), 2 µm NAA (Sigma-Aldrich; Falasca and Altamura, 2003) or 0·1 µm Kin were added to the medium.

Twenty plates (12 × 12 cm, 15–20 seeds/plate) per genotype/line and treatment were placed in the vertical position, at 22 ± 2 °C, under continuous darkness for 14 d, after exposure to white light for 6 h (Takahashi *et al.*, 2003). The plants used as the source of TCLs were grown on a commercial soil starting from seeds prepared as above. To allow stem elongation, 30 plants per genotype/line were grown until reproduction (i.e. day 40 after germination) in a growth chamber, at 22 ± 2 °C, 70 % humidity and long days (white light of 22 Wm^−2^ light intensity).

### Histological analysis of ARs *in planta*, detection of GUS, and GFP epifluorescence

Thirty 14-day-old seedlings of Col and Ws grown under HF conditions were fixed in 70 % (v/v) ethanol, dehydrated by an ethanol series, embedded in Technovit 7100 (Heraeus Kulzer, Germany), longitudinally sectioned at 5 µm with a Microm HM 350 SV microtome (Microm, Germany), stained with 0·05 % toluidine blue and observed under a light microscope.

Stocks of 30 randomly selected *QC25::GUS*, *DR5::GUS*, *PIN1::GUS* and *LAX3::GUS* seedlings per growth medium were harvested at day 14 after sowing, and processed for β-glucuronidase (GUS) staining according to Willemsen *et al.* (1998). Samples of these lines and of their wild type were cleared with chloral hydrate solution (Weigel and Glazebrook, 2002), mounted on microscope slides and observed with Nomarski optics applied to a Leica DMRB microscope. Hypocotyl length was measured under a LEICA MZ8 stereomicroscope before seedling fixation, and AR number was expressed as mean density cm^−1^ (± s.e.). Stocks of 30 randomly selected *pAGL42::GFP* and *pWOX5::GFP* seedlings were harvested on the same day, and green fluorescent protein (GFP) fluorescence was observed under the same microscope equipped with a double wavelength filter set (EX BP 490/20 and BP 575/30; EM BPs 525/20 and 635/40). The images were acquired with a DC500 camera (Leica).

### Thin cell layer culture and microscopic observations

One hundred TCLs (0·5 × 8 mm, six/seven cell layers thick, Supplementary Data Fig. S2A, B) per genotype/line were cultured for 22 d under continuous darkness, at 24 ± 2 °C, on the seedling growth medium to which 10 µm IBA and 0·1 µm Kin were added (Falasca *et al.*, 2004). Forty TCLs of the *sur2-1* mutant and of its wild type (Ws) were cultured either under the latter hormonal condition or under HF conditions.

Ten TCLs per genotype/line were harvested periodically for histological analysis in bright field (the wild type and GUS marker lines) and in epifluorescence (GFP lines). After the GUS assay, *QC25::GUS*, *DR5::GUS*, *PIN1::GUS* and *LAX3::GUS* explants were fixed and embedded as for wild-type seedlings, longitudinally sectioned at 13 µm and observed under light microscopy. *pAGL42::GFP* and *pWOX5::GFP* TCLs were observed using the Leica DMRB epifluorescence microscope. *pAGL42::GFP* TCLs were also observed under a Leica TCS-SP5 confocal microscope after counterstaining with propidium iodide (PI) at 10 mg L^−1^ for 5 min, evaluations being performed by argon laser (EX 488 nm), and detected by LAS software (Leica) with an LP 560 nm filter for PI and with a BP 525/20 nm filter for GFP. Sections (4 µm) of the wild type and *pAGL42::GFP* and *pWOX5::GFP* were also stained with 0·05 % (w/v) toluidine blue for light microscopy observations.

### *YUCCA6 in situ* hybridization

RNA *in situ* hybridization on whole-mount 14-day-old Col and Ws seedlings, grown under HF conditions and continuous darkness, was performed according to García-Aguilar *et al.* (2005). Ten randomly selected seedlings per ecotype were fixed and stored according to Hejátko *et al.* (2006). The samples were treated with digoxigenin-labelled *YUCCA6* antisense and sense RNA probes overnight at 55 °C. *YUCCA6* mRNA detection was performed with 4-nitro blue tetrazolium chloride (NBT)/5-bromo-4-chloro-3-indolyl-phosphate (BCIP) overnight at room temperature. The probe was generated by *in vitro* transcription according to the DIG RNA Labeling Kit instructions (SP6/T7; Roche). cDNA used for probe transcription was synthesized using 5′-CAAACACAACGCTTATCTCTC-3' and 5′-GTAAACTAGCACATGACCACC-3′ as primers.

Hypocotyls from ten HF-grown seedlings of Col and Ws, and ten TCLs of *sur2-1*, Ws and Col cultured on HF and IBA + Kin media, were either processed as for whole-mount hybridizations or fixed in 0·5 % (w/v) glutaraldehyde and 3 % (w/v) paraformaldehyde in phosphate-buffered saline (PBS), dehydrated in an ethanol series, embedded in resin and sectioned (6 µm) for hybridization on sections. Sections were incubated overnight with *YUCCA6* antisense and sense probes at the same concentration and temperature as for the whole mounts, and processed according to Takechi *et al.* (1999) with minor modifications. Sections were observed under light microscopy, and the absence of hybridization signal in the sense probe-treated materials was verified.

### Cytokinin immunolocalization

Thirty 14-day-old seedlings of Col grown under HF conditions were processed and sectioned as for *YUCCA6* hybridization in resin-embedded sections, and the sections were incubated overnight with 1 % *trans-*zeatin riboside primary antibody (OlChemlm Ltd, Czech Republic) in PBS at 4 °C, then with 1 % secondary antibody (Anti-Rabbit IgG, Sigma) with alkaline phosphatase activity for 3 h at room temperature, and finally treated according to Caboni *et al.* (2002). Sections were observed under light microscopy. Control sections were incubated without the primary antibody, and the absence of any immunostaining was verified.

### Statistical analysis

Data were expressed as mean values (± s.e.). Fasciated ARs were counted as single ARs, and their number was expressed as a mean value (±s.e.) on the samples showing fasciation. One-way or two-way analysis of variance (ANOVA, *P* < 0·05) was used to compare effects of treatments and genotypes, and, if ANOVA showed significant effects, Tukey's post-test was applied. Alternatively, Student's *t*-test was used where appropriate (GraphPad Prism 6·0). All the experiments were repeated three times during 2 years, and similar results were obtained (data of the replicates of the second year are shown).

## RESULTS

### AR development *in planta* occurs like LR development, and IBA + Kin treatment enhances the process

**Fig. 1.**
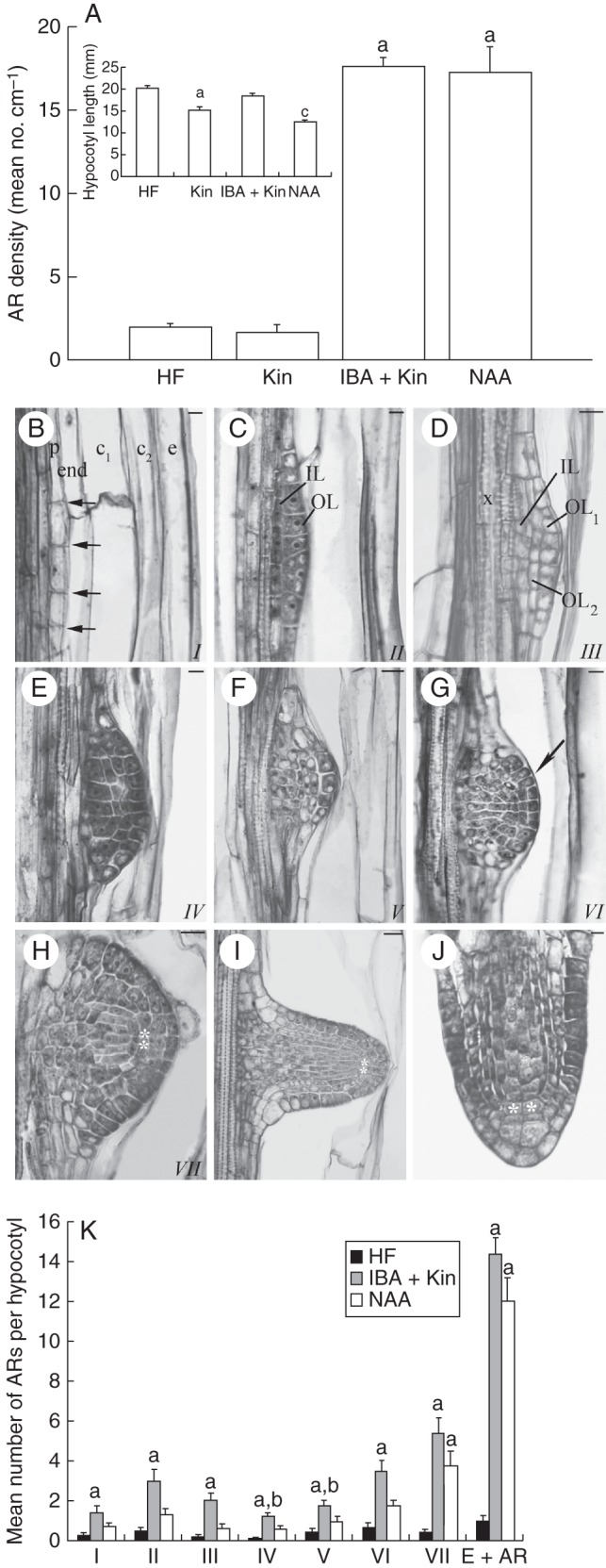
(A) AR mean density (± s.e.) in the hypocotyl of 14-day-old wild-type seedlings grown on HF, Kin (0·1 µm), IBA (10 µm) + Kin (0·1 µm) and NAA (2 µm) media, and hypocotyl mean length (inset). (B–J) Developmental stages of ARs in wild-type seedlings grown on HF medium. (B) First anticlinal divisions in the hypocotyl pericycle (arrows). (C) Outer (OL) and inner (IL) layers formed by periclinal divisions in the cells originated by the first anticlinal divisions. (D) OL periclinal doubling. (E) IL doubling, leading to a four-layered ARP. (F) ARP dome establishment. (G) ARP dome with protoderm specification (arrow). (H) Stage VII ARP showing cells with LR QC morphology (asterisks). (I) Developed ARP emerging from the hypocotyl (QC shown by the asterisks). (J) Apex of a mature AR (QC shown by the asterisks). (B–J) Histological longitudinal radial sections stained with toluidine blue (Ws ecotype). (K) Mean number (± s.e.) of ARs at different stages in wild-type seedlings grown on HF, IBA (10 µm) + Kin (0·1 µm) and NAA (2 µm) media. (A and K) ^a,c^*P* < 0·01 differences from other treatments; ^b^*P* < 0·05 difference between IBA + Kin and NAA. Columns with the same letter are not significantly different. *n* = 30 (Col ecotype). Scale bars: (B–G, J) = 10 µm; (H) = 20 µm; (I) = 30 µm. *I–VII*, developmental ARP stages, p, hypocotyl pericycle; x, protoxylem; c_1_–c_2_, cortex; end endodermis; e, epidermis.

Treatments with exogenous NAA and Kin alone caused a significant reduction in hypocotyl length in comparison with the HF treatment, with the highest reduction under NAA (Fig. 1A, inset). In contrast, the treatment with IBA + Kin did not cause significant changes in comparison with HF treatment (Fig. 1A, inset). The AR density was very low with Kin alone and under HF treatment, but became many-fold higher with IBA + Kin and with NAA (Fig. 1A).

The AR development was histologically investigated in the hypocotyl of 14-day-old HF-grown seedlings of Col and Ws ecotypes. In both ecotypes, the process followed seven stages before AR emergence, as exemplified by the Ws ecotype in Fig. 1B–H. The stages are indicated by Roman numbers, following the numbering proposed by Malamy and Benfey (1997) for LR developmental stages. The pericycle founder cells divided anticlinally (stage I, Fig. 1B), and then periclinally (stage II), forming an outer (OL) and an inner (IL) layer of derivative cells (Fig. 1C). When OL and IL cells expanded, the shape of the AR primordium (ARP) began to appear. At stage III, the OL again divided periclinally, giving rise to an ARP composed of three superimposed layers (Fig. 1D). At stage IV, the IL also divided (Fig. 1E). Anticlinal divisions in the central cells of the *de novo* formed layers occurred at stage V, and the ARP acquired a prominent dome shape (Fig. 1F). The ARP specified its protoderm soon after (stage VI, Fig. 1G). At stage VII, cells with a morphology similar to the QC cells in LR primordia (Malamy and Benfey, 1997) appeared in the ARP dome (Fig. 1H, asterisks). After the QC was established, the ARP rapidly protruded throughout the hypocotyl (Fig. 1I), and elongated, giving rise to a mature AR with a complete apex maintaining the QC position and features (Fig. 1J).

All stages of ARs were in present in similarly very low numbers in HF- and Kin-grown seedlings, as shown for HF in Fig. 1K, whereas their number increased greatly with both auxin treatments, and significantly more with IBA + Kin than with NAA (Fig. 1K). In contrast to the alternating pattern characterizing the appearance of the HF-formed ARs, opposite ARs appeared with both auxin treatments, but mainly with NAA (Supplementary Data Fig. S1A, B). ARs with a double tip (i.e. fasciated ARs, Supplementary Data Fig. S1C) also appeared, but their presence was sporadic with IBA + Kin, whereas they occurred in about 50 % of the seedlings, and with a mean number of 1·8 (±0·4), under the NAA treatment.

### PR/LR QC markers are expressed in the AR QC *in planta*, but *WOX5* is also expressed in the early derivative cells

**Fig. 2.**
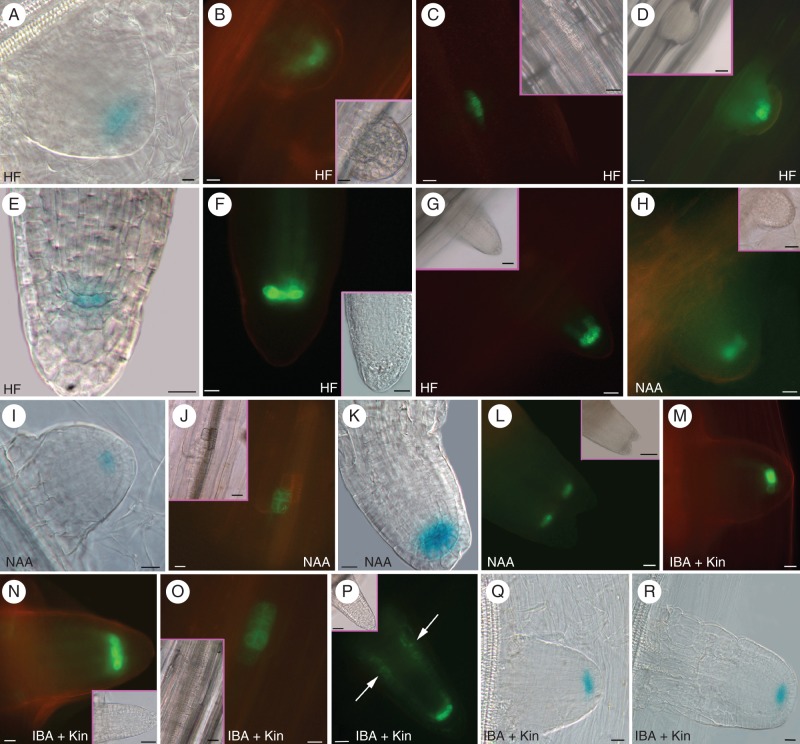
Expression of PR/LR QC markers during AR formation in Col seedlings grown for 14 d under various hormonal treatments. (A–G; HF) *QC25::GUS* (A), *pAGL42::GFP* (B) and *pWOX5::GFP* (D) in the QC at stage VII, and *pWOX5::GFP* at stage II (C). *QC25::GUS* (E), *pAGL42::GFP* (F) and *pWOX5::GFP* (G) in the QC, and lateral initials (G) of emerged ARs. (H–L; NAA) *pAGL42::GFP* (H) and *QC25::GUS* (I) in the QC of stage VII ARPs. *pWOX5::GFP* at stage II (J), *QC25::GUS* at the tip of a regular AR (K) and *pAGL42::GFP* in the twin tip of a fasciated AR (L). (M–R; IBA + Kin) *pAGL42::GFP* in the QC of not yet emerged (M) and emerged (N) ARPs. *pWOX5::GFP* at stage II (O), and in the QC, lateral initials and pericycle cells forming LRs (arrows) in emerged ARPs (P). *QC25::GUS* in the QC at stage VII (Q), and in emerged ARPs (R). Insets in fluorescence pictures show corresponding bright-field images. Scale bars: = (A, B and inset, E, F, H–K, M–O, R) 10 µm; (C, D, G, L, P–Q, and insets in C, F, J, N–O) = 20 µm; (insets in D, G, H) = 30 µm; (inset in P) = 40 µm; (inset in L) = 100 µm.

The analysis of *QC25::GUS* and *pAGL42::GFP* HF-grown seedlings revealed that the cells of stage VII ARPs resembling the LR QC cells (Fig. 1H, asterisks) expressed both these QC markers (Fig. 2A, B), and QC identity was confirmed by the expression of the *pWOX5::GFP* construct (Fig. 2D), another PR/LR QC marker. However, *WOX5* was active from stages I–II (Fig. 2C). *QC25*, *pAGL42* and *WOX5* continued to mark the QC in the protruding ARs (Fig. 2E–G). Also in the seedlings grown with NAA and IBA + Kin *pAGL42::GFP* and *QC25::GUS* signals appeared at stage VII (Fig. 2H, I, M, Q), and *WOX5::GFP* before, i.e. around stage II (Fig. 2J, O), and all markers maintained expression in the QC of protruding/mature ARs (Fig. 2K, N, P, R). The markers were even expressed at the tip(s) of the fasciated ARs formed in the NAA treatment (Fig. 2L).

### Auxin accumulation precedes AR formation, and an auxin maximum is established at the ARP tip and maintained in the AR apex

**Fig. 3.**
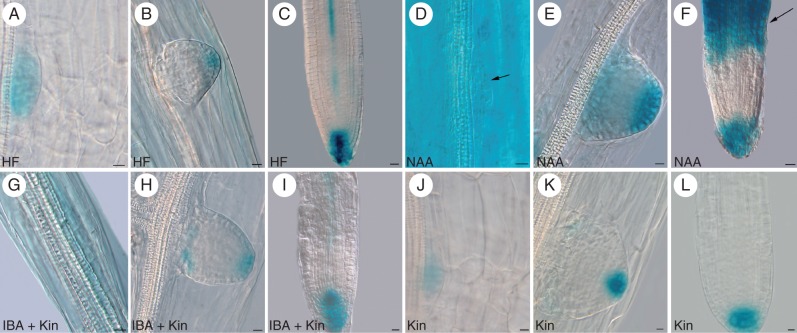
*DR5::GUS*, monitoring IAA presence in developing ARs from 14-day-old Col seedlings grown under different hormonal treatments. (A–C; HF) *DR5* in a stage IV ARP located at the hypocotyl transition zone (A), in the tip of a stage VII ARP (B) and in the QC, flanking initials, columella and developing vasculature of a mature AR (C). (D–F; NAA) Strong *DR5* at stage III (arrow) and in surrounding hypocotyl cells (D), at stage VII (E) and in the QC, surrounding initials, cap and differentiated tissues (arrow) of a mature AR (F). (G–I; IBA + Kin) *DR5* at stage II (G), at the base and tip of a stage VII ARP (H) and in the QC, surrounding initials, columella and developing vasculature of a mature AR (I). (J–L; Kin) Faint *DR5* signal in a stage III ARP located at the transition zone (J), stronger expression at the tip of a stage VII ARP (K) and in the QC, surrounding initials and columella of a mature AR (L). Scale bars: (A, B, D, E, G–L) = 10 µm; (C, F) = 20 µm.

The histochemical analysis of the *DR5::GUS* seedlings grown without exogenous hormones showed that GUS staining, monitoring the presence of IAA, occurred in the vascular parenchyma of the transition zone before ARP formation (Supplementary Data Fig. S1D). Staining also occurred in the founder cells, and their derivatives, independently of the treatment (Fig. 3A, D, G, J); however, the staining was very faint in the presence of exogenous cytokinin alone (Fig. 3J). In the HF-growing seedlings, an IAA maximum was established to occur in the tip of stage VII ARPs (Fig. 3B), and was maintained in the QC, flanking initials and cap cells of the mature AR apex (Fig. 3C). Auxin was also present in the AR vasculature (Fig. 3C). The exogenous NAA caused an enhancement of staining during the entire process (Fig. 3D–F). Moreover, in the mature ARs, tissue differentiation occurred very near the tip, and the differentiated tissues were stained (Fig. 3F, arrow). Under IBA + Kin, the *DR5* signal was more similar to HF than NAA treatment, in terms of both localization and intensity (Fig. 3G–I). Up to stage III (Fig. 3J), the pattern of GUS staining under Kin alone was weaker than under IBA + Kin, and even HF, and no signal was present in the hypocotyl vasculature. However, an auxin maximum was generated at stage VII (Fig. 3K), and maintained in the tip of the mature AR (Fig. 3L).

### AR development *in planta* is related to the expression of *PIN1* auxin efflux and *LAX3* auxin influx genes

**Fig. 4.**
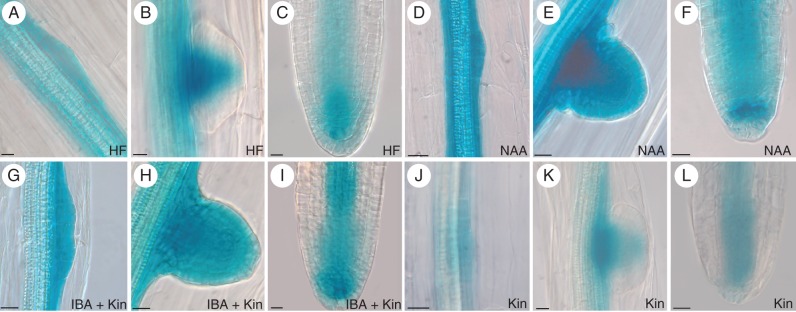
*PIN1::GUS* expression in developing ARs from 14-day-old Col seedlings grown under different hormonal treatments. (A–C; HF) *PIN1* expression at stage III (A), at the base and in the middle of a stage VII ARP (B) and in the niche, cap and procambium of a mature AR (C). (D–F; NAA) High *PIN1* expression at stage III (D), at stage VII (E) and in the whole apex of a mature AR (F). (G–I; IBA + Kin) High *PIN1* expression at stage III (G) and VII (H), and in a mature AR tip, except for the differentiating cortex and epidermis (I). (J–L; Kin) Very weak *PIN1* expression at stage I (J), higher expression at the base and middle part of a stage VII ARP (K) and faint expression in the niche and procambium of a mature AR apex (L). Scale bars: (A,B) = 10 µm; (C–L) = 20 µm.

The expression of the auxin efflux regulator *PIN1*, involved in generating auxin maxima in PR and LRs (Petrášek and Friml, 2009), was examined during AR development *in planta*. *PIN1* was expressed in the hypocotyl vasculature and in the surrounding cells derived from the pericycle AR founder cells, independently of the treatment (Fig. 4A, D, G, J). However, in comparison with HF treatment (Fig. 4A), the signal was reinforced by the treatments with the exogenous auxins (Fig. 4D, G), and reduced by that with cytokinin alone (Fig. 4J). At stage VII, the signal was shown by the entire ARP, under both auxin treatments (Fig. 4E, H), and by its basal and middle parts under HF, and under Kin alone, in particular (Fig. 4B, K). Expression was also detected in the AR tip, i.e. niche and surrounding derivative cells, procambium and cap cells, and, again, at higher levels in the presence of the exogenous auxins (Fig. 4F, I) in comparison with HF, and with Kin alone, in particular (Fig. 4C, L). Moreover, expression was turned off in the differentiating cortical and epidermal cells of the ARs (Fig. 4C, I, L), except that in the NAA treatment (Fig. 4F).

**Fig. 5.**
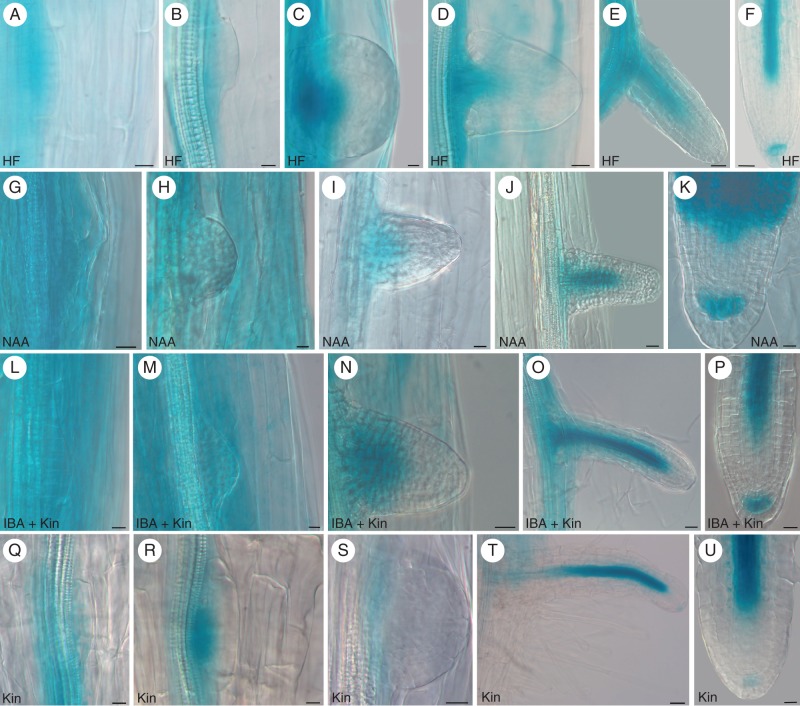
*LAX3::GUS* expression during AR development in 14-day-old Col seedlings grown under different hormonal conditions. (A–F; HF) Expression at stage III (A) and IV (B), at the base of a stage VII ARP (C), in the forming vasculature of emerging (D) and elongating ARPs (E) and in a few cap cells, and the differentiating vasculature of a mature AR (F). (G–K; NAA) High and uniform expression at stage IV (G) and VI (H), in the ARP basal half, before (I) and after (J) protrusion, and in cap cells and precociously differentiated tissues in a mature AR (K). (L–P; IBA + Kin) High expression at stage III (L) and V (M), in the basal half of an emerging ARP (N), in the vasculature of an elongating ARP (O) and a mature AR (P), and in some cap cells in the latter (P). (Q–U; Kin, hypocotyl transition zone) *LAX3* expression at stage II (Q) and IV (R), at the base of a stage VII ARP (S), in the developing vasculature of elongating ARPs (T) and mature ARs (U), and faintly in the AR cap (U). Scale bars: (A, B, H, I, K, M, Q–S) = 10 µm; (D, G, L, N–P, U) = 20 µm; (C, E, F, J, T) = 30 µm.

In the HF treatment, *LAX3::GUS* expression was weak in the hypocotyl vasculature, but increased corresponding to the derivatives of the pericycle AR founder cells (Fig. 5A). Expression was reinforced at early ARP stages (Fig. 5B), becoming progressively stronger in the basal–middle portion of the developing ARP, and appearing in the surrounding hypocotyl peripheral tissues (Fig. 5C, D). From ARP emergence onwards, *LAX3* signal also marked the ARP vasculature, extending up to the elongation zone (Fig. 5E). The apical meristem was without signal (Fig. 5E); however, in the mature AR tip, the signal appeared in the cap (Fig. 5F). Under the auxin treatments, expression was strongly detected in the hypocotyl, early AR stages and ARPs (Fig. 5G, H, L, M). From ARP emergence onwards, the expression pattern did not change in comparison with the HF treatment (Fig. 5I, J, N, O), with *LAX3* continuing not to be expressed in the apical meristem (Fig. 5J, O). In the AR tip, the expression was again shown in the cap, and more extensively under NAA than under IBA + Kin (Fig. 5K, P). In the former treatment, the precociously differentiated tissues also showed expression (Fig. 5K). Under treatment with Kin alone, the hypocotyl vasculature showed expression at the transition region only. ARPs elongated in this region only, and the expression pattern during their development recapitulated that observed under HF, but with a weaker signal (Fig. 5Q–U).

### The ARs formed *in vitro* express the same QC markers of the ARs *in planta* and, similarly, *WOX5* is expressed from the first divisions

**Fig. 6.**
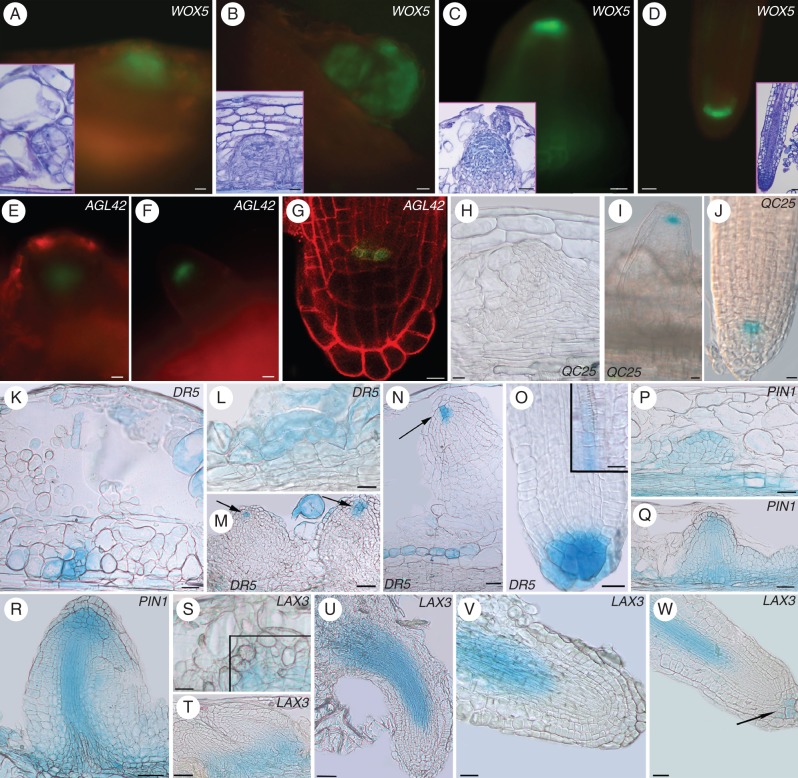
Expression of QC markers (A–J), auxin monitoring (K–O), and auxin efflux (P–R) and influx (S–W) gene expression during AR formation in IBA + Kin-cultured TCLs. (A, B) *WOX5* expressed in meristematic cell clusters (A) and root meristemoids (B). Corresponding light microscopy images are shown in the insets. (C, D) Emerging ARPs (C) and apices of elongated ARs (D) showing *WOX5* in the QC and lateral initials. Corresponding light microscopy images are shown in the insets. (E) Early-domed ARPs showing the appearance of *pAGL42*. (F) Emerging ARPs with *pAGL42* expression in the QC. (G) Confocal microscopy image of mature AR apices with *pAGL42::GFP* signal in the QC. (H) No expression in early-forming ARPs of *QC25::GUS* TCLs, but expression in the QC of emerged ARPs (I) and elongated ARs (J). **(**K) Meristematic cell clusters with *DR5::GUS* expression. (L) Detail of a meristemoid showing signal. (M, N) Not yet protruded ARPs showing *DR5* expression in the tip (arrows in N). (O) *DR5::GUS* expression in the niche and cap of mature ARs. The inset shows expression in the forming vasculature. (P) *PIN1* expression in early-domed ARPs, corresponding to stage VII *in planta*, and endodermis-derived cells at the base. (Q) Not yet emerged ARPs showing *PIN1* signal, mainly in the tip and forming vasculature. (R) Protruded ARP with *PIN1* expression in the vasculature, procambium and apex. (S) *LAX3* expression in forming meristemoids (square). (T) Not yet protruded ARPs with *LAX3* expression at the base. (U, V) Elongating ARPs after protrusion, with *LAX3* expression in the vasculature, but not in the apex. (W) Mature AR with *LAX3* signal in the developing vasculature and some cap cells (arrow). Insets in A–D, toluidine blue section staining. Scale bars: (insets in A and C, G) = 10 µm; (A, C–F, H, J, O, inset in O, V, W) = 20 µm; (inset in B, I, T) = 30 µm; (inset in D, K–M, S, U) = 40 µm; (N, P–R) = 50 µm; (B) = 100 µm.

Wild-type TCLs formed ARs only in the presence of IBA + Kin, confirming previous results (Falasca *et al.*, 2004). Meristematic cell clusters originated from the stem endodermis (Fig. 6A, inset; Supplementary Data S2B, C), and formed root meristemoids (Fig. 6B, inset). Root meristemoids domed and grew into ARPs, which opened their way through the explant cortex (Fig. 6C, inset) and protruded from the explant epidermis, finally becoming mature ARs (Fig. 6D, inset). Fasciated ARs (Supplementary Data Fig. S2E) appeared on about 20 % of the explants, with a mean number of 2·08 (±0·4) per explant.

*WOX5* was expressed early (days 5–7), marking meristematic cell clusters and meristemoids (Fig. 6A, B). Expression continued in the protruding ARPs, marking the QC, and the lateral initials (Fig. 6C). After 14 d of culture, the first elongated ARs were observed, and showed *WOX5* signal in the QC and lateral initials in the apex (Fig. 6D). Expression was also shown by the apices of the fasciated ARs (Supplementary Data Fig. S2F).

*pAGL42* expression began to be observed later than that of *WOX5*, i.e. in the early-domed ARPs, exhibiting a developmental stage comparable with stage VII ARPs *in planta* (Fig. 6E). In protruding ARPs, and normal/fasciated ARs, *pAGL42* signal marked the QC, as it did *in planta* in the same hormonal treatment and stage (Fig. 6F, G; Supplementary Data S2G). As for *pAGL42*, *QC25::GUS* signal was not present at early AR stages (Fig. 6H), appearing in the QC of the domed ARPs, and continuing to be present in the QC of protruding ARPs and elongating ARs (Fig. 6I, J), as it did *in planta* under the same treatment. *QC25::GUS* was also shown in the tips of the fasciated ARs (Supplementary Data Fig. S2H).

### Auxin accumulation occurs during AR formation in TCLs as well as during AR development *in planta*, and similarly involves *PIN1* and *LAX3* expression

For an in-depth insight into the relationship between ARs formed *in vitro* and *in planta*, IAA accumulation and transport were investigated in IBA + Kin-cultured TCLs excised from *DR5::GUS*, *PIN1::GUS* and *LAX3::GUS* plants.

During the first week of culture, IAA accumulated locally in the stem endodermis-derived meristematic cell clusters (Fig. 6K, L). In the early-domed ARPs, *DR5::GUS* activity became evident in a strict population of the apical cells (Fig. 6M), and this apical localization persisted in the protruding ARPs (Fig. 6N). In the ARs, IAA accumulated in the vasculature (Fig. 6O, inset) and in the apex, i.e. the QC, flanking initials and cap cells (Fig. 6O), defining an auxin maximum, as *in planta* under the same treatment and the HF condition.

*PIN1* was diffusely expressed in the root meristemoids and early staged ARPs (Fig. 6P). The signal intensified in the developing ARPs, starting to mark the procambium (Fig. 6Q). A strong expression along the procambium occurred in the protruded ARPs (Fig. 6R) and ARs, reiterating the pattern observed *in planta* under the same treatment and the HF condition.

*LAX3::GUS* expression was diffuse in the meristemoids (Fig. 6S), like *PIN1*; however, in the forming ARPs, *LAX3* signal became restricted to the ARP base (Fig. 6T), differently from *PIN1*. During further ARP development, expression extended acropetally along the procambium (Fig. 6U). In the elongating ARPs, and in the ARs, the expression was present in the vasculature and procambium up to the elongation zone, but was absent in the apical meristem (Fig. 6V, W), except some cap cells in mature ARs (Fig. 6W), collectively reiterating the *LAX3* expression pattern observed *in planta* under the same treatment.

In contrast to the wild-type TCLs, about 27 % of TCLs from the auxin-hyperaccumulating *sur2-1* mutant produced ARs in the HF medium, and with a mean number of 3·3 (±0·6). The ARs elongated and formed LRs (Supplementary Data Fig. S2I). Under IBA + Kin, ARPs and ARs, arranged in clumps, covered *sur2-1* explants (Supplementary Data Fig. S2J, K). The comparison among treatments and genotypes showed that both the treatment and the genotype significantly (*P* < 0·0001) affected the response, with the *sur2-1* TCLs cultured with IBA + Kin producing an AR number significantly (*P* < 0·0001) higher than wild-type TCLs under the same treatment (i.e. 242·2 ± 22·4 and 93·3 ± 8·8, respectively).

### *Trans*-zeatin riboside immunolocalization occurs concomitantly with *YUCCA6* transcription

**Fig. 7.**
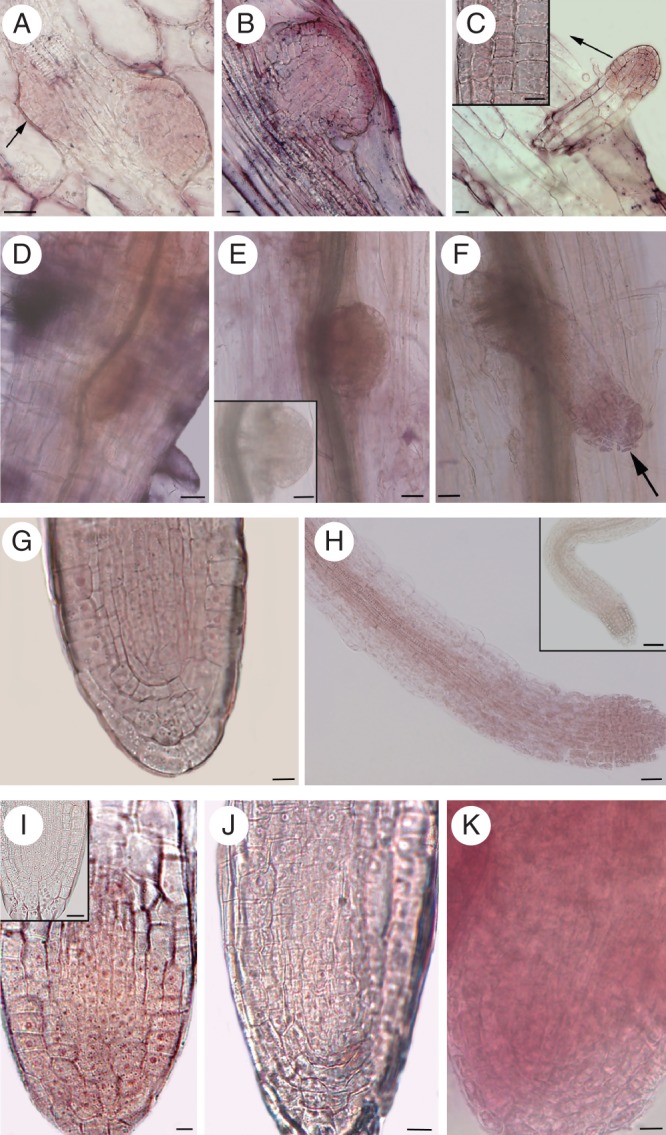
Localization of *trans*-zeatin riboside (A–C) and *YUCCA6* transcription (D–H) during AR development *in planta* [14-day-old Col (A–F) and Ws (G, H) seedlings grown under HF treatment], and *YUCCA6* transcription in ARs from TCLs cultured with/without IBA + Kin (I–K). (A) Stage IV (arrow) and stage VI ARPs showing a diffuse cytokinin immunostaining. (B) ARP just before protrusion showing high cytokinin immunostaining in the protoderm, in particular. (C) Elongating ARP with extensive cytokinin signal at the tip. Differentiating epidermis with staining (arrow) magnified in the inset (longitudinal tangential section). (D–E) *In situ* hybridizations showing *YUCCA6* transcription at stage IV (D) and VI (E). Sense probe control shown in the inset of E. (F–H) *YUCCA6* transcription at the tip (arrow) of protruded ARPs (F), all over the AR apex (G), and in the AR procambium and differentiating vasculature (H). Sense probe control shown in the inset of H. (I) *YUCCA6* transcription in AR apices from IBA + Kin-cultured Ws TCLs. The sense probe control is shown in the inset. (J, K) Low (J) and high (K) *YUCCA6* transcription in the apices of ARs formed by *sur2-1* TCLs cultured without hormones and with IBA + Kin, respectively. (D–F, H, K) Whole-mount RNA *in situ* hybridizations; (G, I, J) RNA *in situ* hybridizations on longitudinal sections of resin-embedded ARs. Scale bars: (inset in C, G, I–K) = 10 µm; (A–C, inset in I) = 20 µm; (D–F, inset in E, H) = 30 µm; (inset in H) = 40 µm.

*Trans*-zeatin riboside, an endogenous cytokinin, was immunolocalized in wild-type seedlings grown under HF conditions. A faint staining appeared in the ARPs around stage IV, and increased in the following stages (Fig. 7A). In the not yet protruded ARPs the immunostaining became strong, marking the dome protoderm, in particular (Fig. 7B). After AR emergence, *trans*-zeatin riboside was immunolocalized in the apex only, marking the differentiating epidermis, in particular (Fig. 7C, inset). Since cytokinin is involved in *PIN* downregulation in the PR (Dello Ioio *et al.*, 2008), the *trans*-zeatin riboside immunolocalization was compared with *PIN1* expression under the same treatment. The results showed that the endogenous cytokinin accumulated preferentially where *PIN1* was expressed at lower levels, both in the ARP (Fig. 4B) and in the AR tip (Fig. 4C).

To verify whether auxin biosynthesis occurred during AR development, possibly to compensate for cytokinin action, *YUCCA6* transcription was evaluated by *in situ* hybridizations in wild-type seedlings and TCLs, and in *sur2-1* TCLs.

In HF-grown seedlings, *YUCCA6* expression was high in the shoot apex and gradually decreased along the hypocotyl up to the transition zone. However, expression appeared in stage IV ARPs, and increased up to stage VII (Fig. 7D, E). The transcription of the gene was reinforced at the tip of protruded ARPs (Fig. 7F), and continued to be present in the apical meristem of the mature ARs, where it also occurred in the procambium and differentiating vasculature (Fig. 7G, H).

In the ARs formed by the wild-type TCLs cultured with IBA + Kin, *YUCCA6* was more expressed than in the ARs of the HF-grown seedlings (compare Fig. 7I and G). The gene was weakly expressed in the apices of the ARs formed by the HF-cultured *sur2-1* TCLs (Fig. 7J), but expression strongly increased in those formed under IBA + Kin (Fig. 7K).

## DISCUSSION

Results show that arabidopsis ARs originating from cells from different tissues, i.e. from the hypocotyl pericycle *in planta* and the stem endodermis in *in vitro* cultured TCLs, similarly establish the QC in the apical meristem, and at the same developmental stage, which corresponds to the stage of QC establishment in the LR primordia. Auxin induces the AR process, and its gradient and biosynthesis sustain QC establishment and maintenance through cytokinin tuning and WOX5 activity.

### An IAA gradient is necessary for AR formation *in planta* and in TCLs

In the pericycle of arabidopsis PR, endogenous auxin accumulates in the founder cells of LRs, and in their early derivatives, and this accumulation is enhanced by exogenous auxin, e.g. by NAA (Benková *et al.*, 2003). The present results verify that early auxin accumulation is a common event in post-embryonic rhizogenesis. In fact, in the hypocotyl of arabidopsis, endogenous auxin accumulated in the vascular parenchyma adjacent to the pericycle founder cells of the ARs, in the latter cells and in their early derivatives. Moreover, the intensity of the auxin signal increased in the presence of NAA and IBA + Kin, whereas it decreased when cytokinin was applied alone. It is known that auxin moves from shoot to root through the hypocotyl vascular parenchyma (Blakeslee *et al.*, 2007). The exogenous auxins might enhance the hormone export from the perivascular cells towards the pericycle cells, increasing the possibility that an auxin content sufficient for AR induction is formed in the latter cells. The *PIN1* auxin efflux carrier is auxin inducible (Vieten *et al.*, 2005). The auxin accumulation in the early derivatives of the hypocotyl pericycle founder cells agrees well with the observed expression of *PIN1* in the same cells, and with the increase in expression caused by the exogenous auxins. This supports that PIN1 might be involved in AR induction, promoting auxin lateral efflux from the hypocotyl vasculature towards the pericycle founder cells. However, other *PIN* genes might also be involved, because the PIN family members are functionally redundant (Blilou *et al.*, 2005). In arabidopsis, the formation of ARs in stem segments cultured *in vitro* is induced by IBA and inhibited by 3,4,5-triiodobenzoic acid, an inhibitor of polar auxin transport (Ludwig-Müller *et al.*, 2005). Moreover, anomalies increase in root-forming NAA-cultured hypocotyl segments when an inhibitor of auxin efflux, i.e. 1-naphthylphthalamic acid, is also applied (Pernisová *et al.*, 2009). These results confirm the well known role of natural/synthetic exogenous auxins for AR induction *in vitro*, but also support that an auxin transport via efflux carriers might be active in the explants. Accordingly, the present results demonstrate that auxin accumulated in the endodermis-derived cell clusters and meristemoids of the AR-forming IBA + Kin-cultured TCLs, with a *PIN1* expression pattern parallel to that observed *in planta*. IBA is a natural precursor of IAA (Strader and Bartel, 2011), and an efficient IBA to IAA conversion is important for arabidopsis LR formation (Strader *et al.*, 2011). It is possible that exogenous IBA is rapidly converted to IAA in the TCL cells close to the medium, and PIN1 directs IAA efflux to the endodermis, inducing in some cells of this tissue an IAA accumulation sufficient for AR initiation. The results obtained with the *sur2-1* mutant, characterized by an endogenous high content of IAA (Delarue *et al.*, 1998), support the hypothesis, because ARs were formed in *sur2-1* TCLs cultured without hormones, whereas AR formation in the wild-type TCLs needs IBA + Kin. In addition, the latter treatment enhanced AR formation in *sur2-1* TCLs, showing an additive function of the exogenous IBA on the endogenous IAA.

LAX (LIKE AUX1) proteins are active in auxin cellular uptake. LAX3 is auxin inducible and a PR stelar marker (Swarup *et al.*, 2008). Under both exogenous auxin treatments (i.e. NAA and IBA + Kin), we observed *LAX3* expression in the hypocotyl vasculature, and a reinforcement of expression in early AR phases. Moreover, *LAX3* and *PIN1* expression patterns were similar during the first AR phases *in planta* and TCLs. Coupling the *DR5*-monitored auxin accumulation with *PIN1* and *LAX3* expression patterns, we believe that a co-ordinated auxin efflux influx, involving the two carriers, caused the IAA gradient essential to the early AR events and building up of the ARP, *in planta* and in TCLs. However, at later developmental stages, *LAX3* and *PIN1* expression patterns differed, i.e. *LAX3* expression was confined at the ARP base, whereas *PIN1* expression extended up to the ARP tip. Also during LR formation, after an initial uniform expression, *LAX3* is excluded by the primordium tip (Swarup *et al.*, 2008).

In addition, LAX3 seemed necessary for AR emergence because it was expressed in the hypocotyl endodermal, cortical and epidermal cells adjacent to the protruding ARP. A similar expression pattern is shown by *LAX3* in the PR during LR emergence, and a relationship between LAX3 and gene expression leading to protrusion has been proposed (Swarup *et al.*, 2008). Moreover, we observed *LAX3* expression in the columella cells. This suggests that the carrier is also necessary for the gene expression required for cell to cell separation in the root cap.

### *WOX5* is an early marker of the AR process

We show that *WOX5* is precociously activated during the AR process, both *in planta* and in *in vitro* culture. In agreement with this, *WOX5* is expressed in the QC precursor cells in the early globular embryo (Haecker *et al.*, 2004), and in the LR founder cells (Ditengou *et al.*, 2008).

Because *WOX5* is induced by auxin (Gonzali *et al.*, 2005), and auxin is present in the LR founder cells (Dubrovsky *et al.*, 2008), auxin has been proposed to specify the QC in the LRs through *WOX5* expression (Ditengou *et al.*, 2008). We can advance a similar hypothesis for the ARs formed both *in planta* and in *in vitro* culture. In fact, based on the expression patterns of *WOX5* and *PIN1* and the observed localization of auxin gradients and maxima, an auxin flow directed towards the tip of the forming ARP might progressively restrict the initial expression domain of *WOX5*, resulting into the apical positioning of the QC at stage VII of development.

### Cytokinin negatively affects *PIN1* and *LAX3* expression

In the PR, the bulk of cytokinin is synthesized in the tip, in the cap in particular, and is exported through the xylem, whereas, in LRs, it is produced in the entire tip and appears not to be transported through the stele (Aloni *et al.*, 2005). We observed that *in planta* exogenous Kin, when applied alone, reduced early auxin accumulation. In PR and LRs, cytokinin is known to regulate auxin negatively by inducing Aux/IAA proteins which downregulate the abundance of PIN proteins (e.g. of PIN1) (Moubayidin *et al.*, 2009; Su *et al.*, 2011). It is possible that a cytokinin-induced reduction of auxin flow occurred at the onset of the AR process to counteract a possible excess in auxin-induced founder cell formation and activity. The relevant immunolocalization of *trans*-zeatin riboside at stage VII suggests a direct role for the hormone also in ARP growth. The cytokinin mainly accumulated in the outermost layers of the ARP, where an almost total absence of *PIN1* expression and auxin accumulation were observed. This suggests that endogenous cytokinin is locally synthesized to downregulate *PIN1* and block the auxin flow in the outermost part of the ARP, forcing PIN1 activity, and auxin flow, to occur along the middle cells up to the ARP tip, here establishing the auxin maximum required for WOX5-related QC positioning. Moreover, the application of cytokinin alone to the seedlings shows that the hormone is also involved in the restriction of the *LAX3* expression domain to the ARP base.

### Local auxin biosynthesis is needed for ARP development

*In planta* the establishment of an auxin maximum in the ARP tip, and its maintenance in the AR, were also observed when kinetin was applied alone to the growth medium. Based on the negative effect of the hormone on the expression of *PIN1* and *LAX3*, it is possible that a local auxin biosynthesis was needed to compensate the cytokinin-reduced auxin flow to the tip and to maintain apical auxin homeostasis and the QC-related gene expression (e.g. that of *WOX5*). In agreement with this, auxin biosynthesis contributes to the auxin homeostasis at the PR tip (Petersson *et al.*, 2009). Moreover, cytokinin is known to promote auxin biosynthesis in PR and LRs (Jones *et al.*, 2010). The present results support the hypothesis, because *YUCCA6* expression and *trans*-zeatin riboside immunolocalization initiated at the same stage (i.e. stage IV), i.e. before QC establishment, increased at stage VII, and were similarly localized in the tip at further stages.

The response of *sur2-1* TCLs is also informative. *YUCCA* genes are not involved in the IAA synthesis pathway which is upregulated by *sur2* mutation (Barlier *et al.*, 2000; Mashiguchi *et al.*, 2011). In agreement with this, *YUCCA6* was weakly expressed in the tip of the ARs formed by the mutant TCLs cultured without hormones. In contrast, expression increased greatly at the tip of the ARPs formed both by *sur2-1* and by wild-type TCLs cultured with IBA + Kin, suggesting that this similar enhancement was directly/indirectly caused by cytokinin application, supporting the regulative role proposed for the hormone on AR formation *in planta*.

**Fig. 8.**
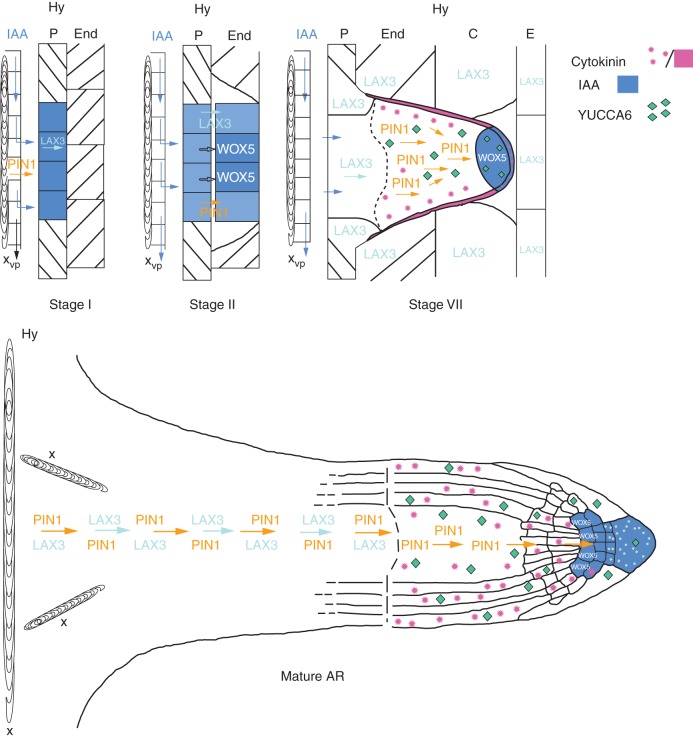
Model of auxin flow, gene expression and cytokinin localization during AR formation *in planta*. At stage I, auxin (IAA) is diverted from the basipetal flow along the vascular parenchyma cells (vp) adjacent to the protoxylem (x) of the hypocotyl (Hy) towards the pericycle (P) cells by PIN1, activating LAX3 and auxin accumulation (blue colour) in the founder cells. At stage II, auxin is maintained in the first-formed inner and outer AR layers by PIN1 (yellow arrow) and LAX3 (light-blue arrows), and *WOX5* is expressed. At stage VII, PIN1 drives auxin flow towards the ARP tip throughout the middle cell files, because cytokinin (pink colour) downregulates *PIN1* in the peripheral ARP layers. Cytokinin also downregulates *LAX3*, limiting the carrier activity at the ARP base (up to the dotted line). The auxin flow driven by PIN1 towards the tip results in an apical auxin maximum, limiting *WOX5* expression at the distal tip, and here establishing the position of the QC. Auxin biosynthesis by YUCCA6 (green diamonds) contributes to auxin maximum positioning in the tip. LAX3 is also active in the Hy endodermis (End), cortex (C) and epidermis (E) around the ARP, possibly favouring protrusion. In the mature AR, the auxin maximum (blue colour) encompasses the QC, flanking initials and cap cells (columella, in particular), and *WOX5* QC expression is maintained. Auxin biosynthesis by YUCCA6 is also maintained (green diamonds), contributing to the persistence of apical auxin accumulation. Also cytokinin is present at the AR tip (pink stars), contributing to the maintenance of auxin homeostasis by a downregulation of *PIN1* in the forming epidermis/cortex, and of *LAX3* in the entire tip, except the cap (light-blue dots). *PIN1* and *LAX3* are expressed in the AR vasculature, and *LAX3* expression stops at the elongation zone border (dotted line).

In conclusion, the ARs organize a QC; its establishment and maintenance are independent of the origin of the founder cells, a co-ordinated action of auxin and cytokinin is needed, and involves expression of specific genes, as proposed in the model of Fig. 8.

## SUPPLEMENTARY DATA

Supplementary data are available online at www.aob.oxfordjournals.org and consist of the following. Figure S1: opposite ARs under NAA and IBA + Kin treatments, fasciated ARs formed under NAA and *DR5::GUS* expression at the hypocotyl–PR transition zone. Figure S2: TCL structure, AR response from wild-type TCLs at day 22 of culture with IBA + Kin, morphology of regular and fasciated ARs, QC marker expression in fasciated ARs and AR-response in *sur2-1* TCLs cultured with/without IBA + Kin.

Supplementary Data
