# Supplementary material for: Auxin and cytokinin control formation of the quiescent centre in the adventitious root apex of arabidopsis
Source: Ann Bot. 2013 Sep 22;112(7):1395–407. doi: 10.1093/aob/mct215 (PMC3806543; doi:10.1093/aob/mct215)
Supplement: Supplementary Data [file supp_112_7_1395__index.html]

Auxin and cytokinin control formation of the quiescent centre in the adventitious root apex of arabidopsis — Auxin and cytokinin control formation of the quiescent centre in the adventitious root apex of arabidopsis — Supplementary Data 

# Auxin and cytokinin control formation of the quiescent centre in the adventitious root apex of arabidopsis

## Supplementary Data

Supplementary Data

**Files in this Data Supplement:**

- Supplementary Data - Pdf file
