# Supplementary material for: Auxin and cytokinin control formation of the quiescent centre in the adventitious root apex of arabidopsis
Source: Ann Bot. 2013 Sep 22;112(7):1395–407. doi: 10.1093/aob/mct215 (PMC3806543; doi:10.1093/aob/mct215)

SUPPLEMENTARY DATA

Fig. S1- (A) Mean percentage of opposite ARs per hypocotyl (±SE) in 14-days-old *Arabidopsis thaliana* Col seedlings grown under continuous darkness either with IBA(10µM)+Kin(0.1µM) or NAA(2µM). ^{a}, $P<0.05$ difference between the treatments (Student's *t*-test). N=30. (B) Detail of the hypocotyl of a seedling grown with NAA showing opposite ARs (red arrows). (C). Dichotomous AR on the hypocotyl of an NAA-grown seedling. (D) Detail of the transition zone between hypocotyl and primary root of a HF-grown *DR5::GUS* seedling showing auxin presence in the hypocotyl vascular parenchyma (blue colour). Bars= 20µm (D); 30µm (C); 100µm (B).

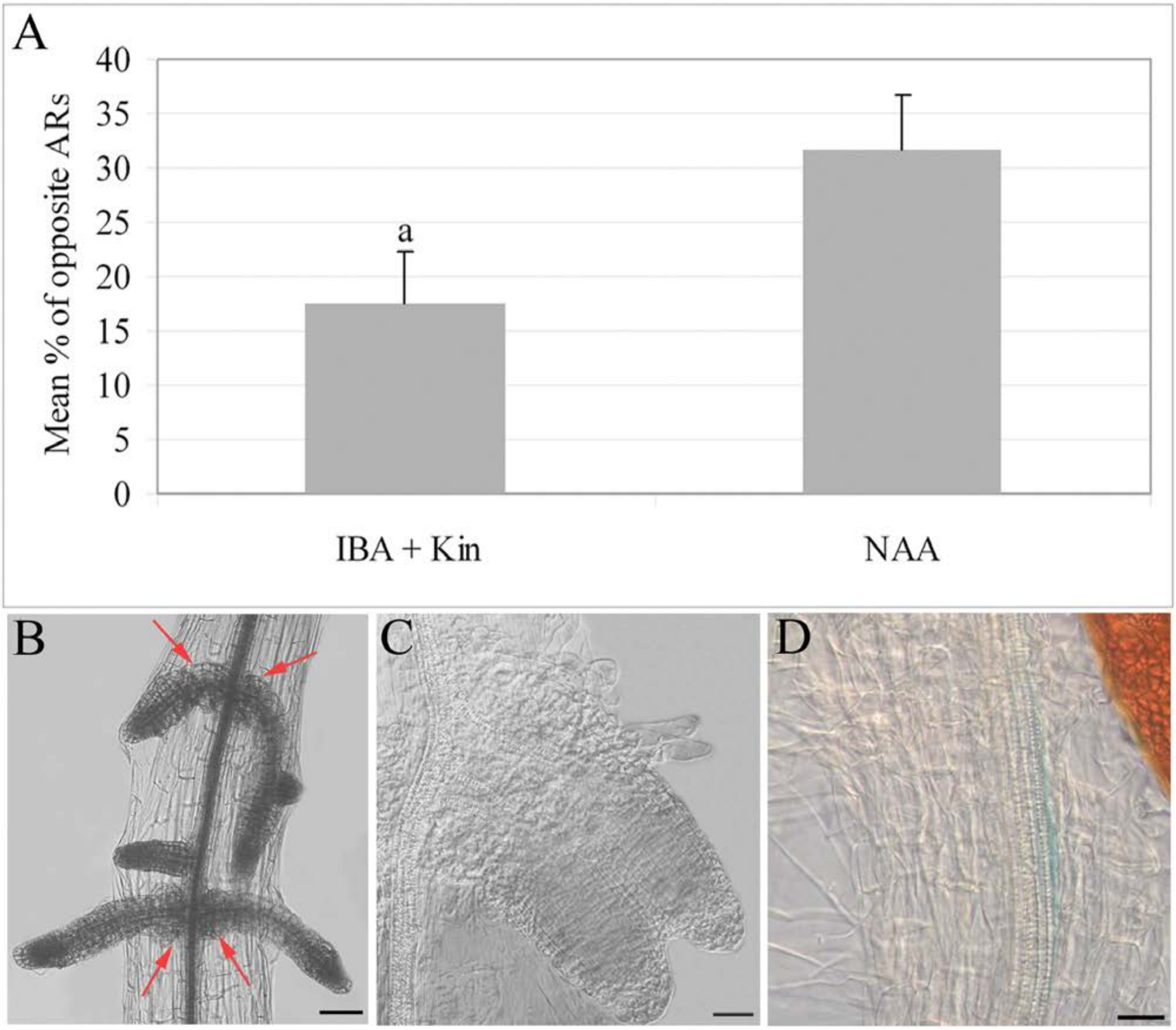

Fig. S2- AR formation from *Arabidopsis thaliana* stem TCLs cultured either with IBA(10μM)+Kin (0.1μM) (A-H, J-K) or without hormones (I) (continuous darkness). TCLs excised from Ws ecotype (A-E), Col ecotype (F-H), and *sur2-1* mutant (Ws background) (I-K). (A-B) Tangential view of a TCL under the stereomicroscope (A) and radial longitudinal section (B) at the excision time.
The uniseriate epidermis (e), three cortical layers, the stem endodermis (end), and the fibers (f) are shown in B. (C) Expanded and separated each other cortical cells and superimposed layers of endodermis derivative cells organizing meristematic cell clusters (arrows) (day 5). (D) TCL with callus and emerged ARPs (arrows) at culture end (day 22) (stereomicroscope image). (E) Example of fasciation: a dichotomous ARP not-yet emerged from the explant epidermis (radial longitudinal section).
(F-H) Twin apices of dichotomous ARs (F-G) and multiple apices of fasciated ARs (H) showing QC-marker signals, i.e., *pWOX5::GFP* (F), *pAGL42::GFP* (G), and *QC25::GUS* (H). (I) Elongated ARs *de novo* formed on HF-cultured *sur2-1*-TCLs. Lateral root formation on these ARs is shown by the arrow. (J-K) *sur2-1*-TCLs cultured with IBA+Kin showing a lot of ARPs emerging from the callus (arrows in J) at culture end. A detail of an ARP is magnified in the Inset of J. Frequent arrangement of *sur2-1*-ARPs in clumps (K). The Insets in the fluorescence pictures show the corresponding bright-field images. (B-C, and E, toluidine blue staining).
Bars= 20μm (B); 30μm (E,F); 50μm (C,H); 60μm (G); 100μm (K, and Insets in F and G); 400μm (Inset in J); 1mm (A,D,I,J).

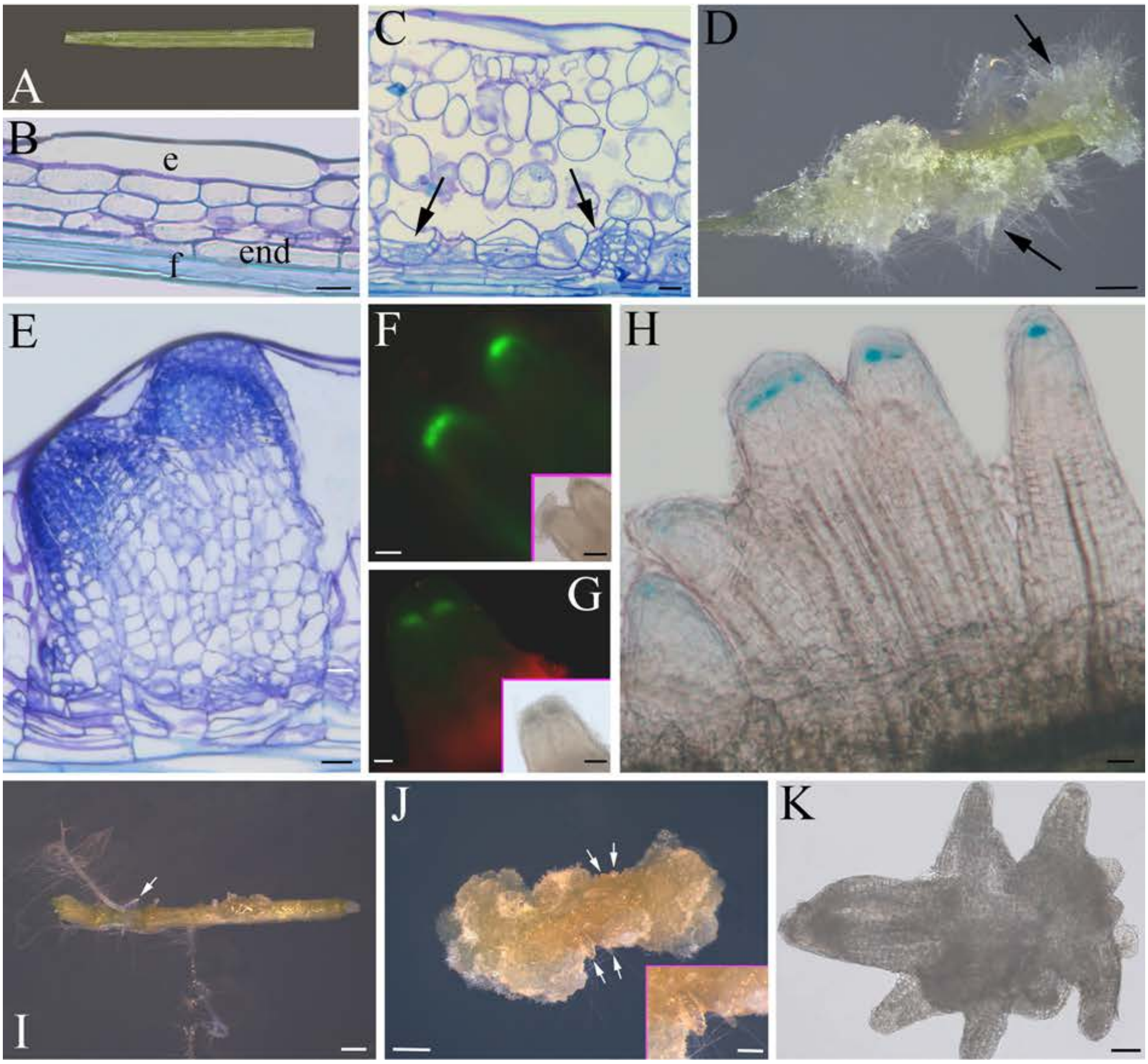

Supplement: Supplementary Data [file supp_mct215_mct215supp.pdf]
